# Worldwide impact of aerosol’s time scale on the predicted long-term concentrating solar power potential

**DOI:** 10.1038/srep30546

**Published:** 2016-08-10

**Authors:** Jose A. Ruiz-Arias, Christian A. Gueymard, Francisco J. Santos-Alamillos, David Pozo-Vázquez

**Affiliations:** 1 Department of Applied Physics I, University of Málaga, Málaga, Spain; 2 Solar Radiation and Atmosphere Modeling Group, Department of Physics, University of Jaén, Jaén, Spain; 3 Solar Consulting Services, Colebrook, NH, USA

## Abstract

Concentrating solar technologies, which are fuelled by the direct normal component of solar irradiance (DNI), are among the most promising solar technologies. Currently, the state-of the-art methods for DNI evaluation use datasets of aerosol optical depth (AOD) with only coarse (typically monthly) temporal resolution. Using daily AOD data from both site-specific observations at ground stations as well as gridded model estimates, a methodology is developed to evaluate how the calculated long-term DNI resource is affected by using AOD data averaged over periods from 1 to 30 days. It is demonstrated here that the use of monthly representations of AOD leads to systematic underestimations of the predicted long-term DNI up to 10% in some areas with high solar resource, which may result in detrimental consequences for the bankability of concentrating solar power projects. Recommendations for the use of either daily or monthly AOD data are provided on a geographical basis.

Both directly and indirectly, the energy received continuously from the Sun sustains all forms of life on Earth. Similarly, during the forthcoming decades, solar energy is expected to play a principal role in the global energy system and in global strategies to mitigate the climate change impacts. Concentrating solar technologies are part of this trend. They use optical systems to concentrate solar irradiance many hundred times to feed a power conversion unit. Consequently, only the solar irradiance coming from a small solid angle centered on the sun (typically ≈1°) can be used in these systems. (This small angle is actually less than the aperture angle of modern pyrheliometers, typically ≈2.5°)^1^. This part of the total solar irradiance is known as direct normal irradiance; it is hereinafter referred to as DNI.

The most important concentrating technologies for utility-scale electricity production are concentrating photovoltaic (CPV)^2^ and concentrating solar thermal power (CSP)^3^. CPV is still in a very early development stage but promises conversion efficiencies never seen so far in conventional photovoltaic systems. In contrast, CSP technologies have been successfully implemented since the 1970s and currently count multiple commercial plants in service. The CSP installed capacity is foreseen to grow worldwide from 4.7 GW currently to ≈1 TW by 2050^4^. Interestingly, a unique advantage of CSP compared to most other renewable energy technologies is that such plants can dispatch power round-the-clock by incorporating a thermal storage system and over-sizing the solar field accordingly. There are suggestions that a geographically dispersed CSP network could be a baseload-capable technology in some parts of the world, which would be crucial for a successful decarbonization of electricity production and mitigation of climate change impacts^5^,^6^,^7^. However, the optimal geographical distribution of CSP and CPV plants can only be achieved through a highly accurate and precise characterization of the long-term surface solar resource and its interannual variability. This long-term characterization is typically based on solar radiation series of yearly sums obtained from sub-daily evaluations (e.g., 15-min data). From such series, the future production of the solar power plant throughout its expected lifetime can be estimated during the feasibility stages of the solar project. It is thus highly important to provide unbiased characterizations of the expected long-term resource that enables an unbiased analysis of costs and revenues. Actually, the project’s bankability depends on the high-confidence demonstration (by the developer) that the foreseen long-term production and revenue are unbiased.

The characterization of the expected long-term solar resource can be best achieved by observing solar radiation with ground-based radiometers. Nonetheless, such stations are still scarce and most often provide data over too short periods. Therefore, since appropriate historic ground records are not usually available at the remote locations suitable for solar exploitation, the long-term DNI availability needs to be evaluated through modelling. This, in turn, creates a potential source of error due to uncertainties in the radiation model inputs. In particular, it is known that, under the cloudless conditions required for CSP plants to generate heat, aerosol optical properties need to be known precisely to guarantee accurate predictions of DNI^8^,^9^.

CSP, being a relatively mature technology with a good track record, offers a good case study to illustrate the importance of accuracy in long-term solar radiation estimates. In general, the financial investment required by solar projects, and particularly CSP projects, is so large that their success largely depends on the availability of unbiased long-term solar datasets that allow attracting competitive financing. This issue is so important that it constitutes a whole body of research known as solar radiation bankability^10^, which investigates methods for data quality enhancement (e.g., data fusion techniques of multiple sources of solar radiation data^11^,^12^) and data quality assurance (e.g., by defining new application-tailored data quality metrics^13^,^14^). Most generally, it is known that biases in the DNI solar resource are the main source of induced uncertainty in the simulated energy output of CSP plants^15^. As a case example, the gross revenue of a reference 50 MW CSP plant in southern Spain—where the long-term mean annual DNI resource is ≈2000 kWh/m^2^—is estimated at ≈30–40 M€/yr. Assuming that its annual production depends linearly on the available annual DNI, a 5% error in the estimated annual DNI availability translates into ≈2 M€/yr deviation in the estimated annual gross revenue. This value, added to other inherent uncertainties in the modelled DNI resource, is high enough to challenge the financial feasibility of the CSP project and thus the optimal exploitation of this technology worldwide.

Various studies^16^,^17^,^18^,^19^,^20^ have shown that DNI is much more sensitive than global horizontal irradiance (i.e., the sum of the direct and diffuse irradiances on a horizontal surface) to changes in the cloud and aerosol amounts because these changes produce counteracting variations in the direct and diffuse components that largely cancel out at making global horizontal irradiance. In parallel to the high DNI sensitivity to cloud and aerosol changes, low-cloudiness conditions are critical here because (i) large solar energy facilities are installed in areas with prevailing cloudless skies to maximize DNI, and (ii) power production can continue under cloudy situations only by discharging the storage system. In particular, in the absence of clouds, aerosol is the key atmospheric constituent that controls most of the attenuation of solar radiation throughout the atmosphere, and thus the resulting surface irradiance. Aerosol loads may reach levels yielding up to ≈70% DNI extinction, or even higher under extreme circumstances, over highly-turbid regions^21^. Consequently, the uncertainty in solar resource assessments over low-cloudiness areas is essentially determined by the uncertainty in the description of the aerosol optical properties. These aerosol properties ultimately determine the solar radiation extinction. Therefore, since aerosol optical properties are typically difficult to measure and highly variable over time and space, the uncertainty in solar resource assessments in the absence of clouds may be high. Such inaccuracies in aerosol data have a detrimental effect on the prediction of daily CSP power output^22^.

The current state-of-the-art approach for the assessment of the long-term solar resource over wide areas relies on relatively accurate algorithms to evaluate cloud extinction based on the cloud reflectance sensed from spaceborne spectrometers^23^,^24^,^25^,^26^,^27^,^28^. This is further combined with clear-sky irradiance evaluations based, most importantly, on aerosol optical depth (AOD) data. Roughly, AOD is the physical magnitude predominantly driving the amount of solar radiation extinction produced by atmospheric aerosols. The historical solar resource over a period of ideally 15–20 years is then derived from these algorithms at continental or global scale. Such a period is a minimum to properly evaluate the interannual variability in DNI, which might be significant^16^,^29^,^30^. However, whereas cloud reflectance has been observed from geostationary satellites for the last two decades at continental and global scales using hourly or sub-hourly time steps, reliable AOD remote sensing retrievals started only in 2000, using multi-wavelength sensors on polar orbiters^31^,^32^,^33^,^34^. Because of these polar orbits, the latency of AOD observations may span up to several days until the satellite swath sweeps the same area again. Even more importantly, AOD retrievals are only feasible under completely cloud-free situations, thus considerably limiting the final amount of valid retrievals. Additionally, some retrieval algorithms cannot work properly, if at all, over high-reflectance surfaces, such as arid areas^33^—precisely where CSP/CPV projects should flourish.

Nowadays, numerical weather prediction models coupled with chemistry transport models can advantageously simulate the major physical processes that aerosols undergo in the atmosphere over space and time. From their predictions, AOD can be evaluated at sub-daily time steps^35^,^36^,^37^,^38^. However, most of these models are still in early development stages and the AOD datasets based on their simulations are scarce, available only for limited historical periods, or with only limited accuracy. Thus, although they have started to be used for solar radiation evaluation in combination with satellite-based retrievals of cloud reflectance^39^,^40^,^41^, they are not expected to replace the traditional approach based on AOD observations.

Overall, due to the paucity of spatio-temporally continuous AOD retrievals, the solar radiation extinction caused by aerosols is usually modelled from AOD datasets with, at best, monthly resolution, or even from climatologies that have the additional drawback of dampening out any aerosol-related interannual variability^42^,^43^,^44^,^45^,^46^.

The impact of AOD on DNI’s extinction is characteristically nonlinear^16^,^47^, as defined by the relationship 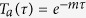, where *T*_*a*_ is the aerosol transmittance and *τ* stands for AOD. (Even though τ is a spectral magnitude, its spectral details are not relevant for this discussion and are omitted in what follows). The “air mass” parameter *m* is used to account for the actual atmospheric optical length; it essentially accounts for the solar position. The extinction’s nonlinearity has important implications for the modelling of DNI from monthly representations of AOD, depending on the singular temporal distribution of AOD at the site of interest. Figure 1a shows the characteristic shape of the direct aerosol transmittance as a function of AOD (black line). Along with this curve, Fig. 1a also shows a typical frequency distribution of daily or sub-daily AOD data at a clean (low-turbidity) site (solid red curve). Interestingly, in this case, the aerosol transmittance is approximately linear over the range of values of the AOD frequency distribution (dashed red line). Mathematically, this can be understood using the following series expansion of the exponential function:


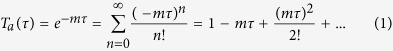


If τ is close to zero, the second and higher-order terms of the series expansion are much lower than the linear term. Then, *T*_*a*_(*τ*) ≈ 1 − *m τ*. As a consequence, in general, a simple relationship exists, *T*_*a*_(*τ*) = *a*_*0*_ + *a*_*1*_ *τ*, for parameters *a*_*0*_ and *a*_*1*_ to be determined for specific local conditions if *τ* is small. The expected value of this transmittance, *E*[*T*_*a*_(*τ*)], can be evaluated from the expected value of *τ*, *E*[*τ*], as *E*[*T*_*a*_(*τ*)] = *a*_*0*_ + *a*_*1*_ *E*[*τ*] provided that *a*_*0*_ and *a*_*1*_ do not depend on *τ*. Therefore, if *τ* is small, the impact of AOD on the monthly-mean DNI at that site is expected to be reliably modelled from only the monthly-mean AOD value, which in turn can be easily extracted from a monthly AOD dataset.

Consider now the particular AOD data distribution shown in Fig. 1b (solid green curve), in which the AOD values span a much wider range. This is the typical case at high-turbidity sites. The DNI extinction curve (solid black line) over the variability range of AOD values cannot at all be approximated using a linear function anymore (dashed green line). Hence, the previous simplifications regarding *E*[*T*_*a*_(*τ*)] for low-turbidity sites cannot apply here. This situation necessarily results in biased estimates of the mean DNI if only monthly-mean AODs (i.e., *E*[*τ*]) are used as in the low-turbidity case.

To better understand the origin of this bias, let now write the aerosol extinction *T*_*a*_(*τ*) using its series expansion as shown in equation (1), i.e., *T*_*a*_(*τ*) = *a*_*0*_ + *a*_*1*_ *τ* + *…* + *a*_*n*_ *τ*^*n*^, for parameters *a*_*0*_*, a*_*1*_*… a*_*n*_ to be determined according to the local conditions. The expected value of the aerosol transmittance is *E*[*T*_*a*_(*τ*)] = *a*_*0*_ + *a*_*1*_ *E*[*τ*] + *…* + *a*_*n*_ *E*[*τ*^*n*^], which clearly does not depend on *E*[*τ*] alone. This explicitly shows that the origin of the bias is in neglecting the expected value of the second and higher-order terms of the series expansion.

As can be interpreted from Fig. 1, the DNI bias that would result from using monthly-mean AOD data depends on both the range of AOD values (i.e., the width of the AOD data distribution) and the shape of the aerosol transmittance curve over this range of AOD values. The former strongly depends on the AOD temporal variability and is also affected by its representation time scale. From now on, representation time scale (or simply, time scale) refers to the temporal resolution of the underlying AOD time series. Thus, for instance, a daily representation time scale refers to daily time series of AOD or its frequency distribution. Ultimately, both the AOD frequency distribution and the shape of the aerosol transmittance curve depend on the particular chemical speciation of aerosols and their size distribution, which determine their optical properties, including AOD, most importantly.

## Methods

The present section describes the methodology used to estimate the global-scale systematic bias that results in the long-term DNI prediction because of the use of temporal representations of AOD in the range 1–30 days.

### Reference approach based on high-quality ground AOD observations

The variation of the long-term DNI as a function of the AOD time scale is evaluated using as skill reference the long-term DNI which is obtained from daily AODs. Specifically, the associated relative deviation is evaluated as 100 × (*B*_*n*_ − *B*_*1*_)/*B*_*1*_, where *B*_*n*_ is the long-term DNI calculated using *n*-day AOD representations and *B*_*1*_ is the long-term DNI obtained from 1-day AODs. Thus, a negative value means an overall underestimation of long-term DNI with respect to *B*_*1*_. The results using daily AODs are considered a reference because the most important processes driving aerosols in the atmosphere occur at synoptic scales spanning periods of only a few days. Typical examples are dust storms in desert areas, atmospheric circulation blocking (air stagnation) and frontal activity or seasonal biomass-burning episodes. At sub-daily scales, the variability of aerosols is strongly explained by local constraints (e.g., topography and human activities). Since considering such local effects would largely hamper the generalization of results, they are specifically excluded from this study. In addition, a daily representation of AOD is currently seen as sufficient by the solar industry. A numerical justification of the selection of the daily time scale as a skill reference directly stems from the results in Supplementary Table S1 and companion discussion online. This supplement provides an estimate of the extent to which the sub-daily AOD variability and its impact on DNI can be neglected with respect to the expected variability and impact of AOD on DNI at synoptic scales.

The daily AOD ground data used here are compiled from multi-year time series of cloud-filtered (“Level 2”) observations at 15-min resolution provided by 214 stations of the Aerosol Robotic Network (AERONET)^48^. (A complete list of the AERONET sites is provided in Supplementary Table S1). This worldwide network offers high-quality AOD observations that are widely considered the ultimate reference to validate other sources of AOD data. The method consists of two steps: (1) calculation of the characteristic frequency distribution of AOD at different time scales from daily to monthly with 1-day time steps (see Supplementary Fig. S1); and (2) a Monte-Carlo approach to estimate the long-term DNI values at each AOD time scale and site based on the evaluations of a high-performance clear-sky solar radiation model.

In the first step, new AOD observations at 30 different time scales from daily to monthly are generated using a moving-average process over the original AERONET dataset with averaging window sizes varying from daily to monthly at 1-day time steps. These observations are used to generate AOD frequency distributions at every location for each one of the 30 time scales (for an example, see Supplementary Fig. S1). The moving-average approach is chosen because it maximizes the number of resulting samples (as opposed to a slicing approach, for instance) from which the AOD frequency distributions are generated. In general, the mean of the AOD distributions remain approximately constant regardless of time scale. Conversely, the distribution’s standard deviation decreases monotonically as time scale gets coarser, with greater decays where the temporal variability of AOD is greater (See Supplementary Fig. S1).

In this study, standard deviation is preferred over variance because it has same units as mean, thus its relative size with respect to the mean is more easily judged. Since these observations are obtained from a moving-average process, they are expected to be autocorrelated. As a result, the standard deviation calculated from them is only a biased estimation of the true standard deviation. However, this effect is here corrected and the calculated standard deviations constitute a better representation of the true values^49^.

In the second step, 3 × 10^4^ random samples are drawn from each AOD frequency distribution. These samples are used to compute DNI with the high-performance Reference Evaluation of the Solar Transmittance 2-band (REST2) clear-sky solar radiation model^50^. The 3 × 10^4^ resulting DNI values are averaged to get an estimate of the long-term DNI resource. The standard deviation is also computed. This high number of samples (which was determined after a preliminary sensitivity study) guarantees the reliability of the long-term value thus obtained. However, in order to further reduce the statistical noise, this process is repeated 30 times for each time scale. The long-term mean and standard deviation DNI values are finally obtained by taking the mean of the 30 repetitions. This overall process is repeated independently for each time scale from daily to monthly.

REST2 has been shown to be well suited for an extremely wide range of turbidity conditions, from very low to very high AOD values^8^. It is a deterministic model that simulates the incoming shortwave clear-sky solar irradiance at the surface in two separate spectral bands (0.29–0.7 μm and 0.7–4 μm), assuming a simplified single-layer cloudless atmosphere. It requires solar zenith angle, atmospheric pressure at the surface, precipitable water, total column ozone, Ångström turbidity parameter (i.e., AOD at 1 μm), Ångstrom’s exponent and, optionally, aerosol single-scattering albedo and aerosol asymmetry parameter. Since REST2 requires the Ångström turbidity parameter, AOD at 1 μm from AERONET is used in the moving-average processes described above, from which the AOD frequency distributions are generated. The aerosol single-scattering albedo and asymmetry parameter are set to their default values (0.92 and 0.70, respectively). This simplification is perfectly justified here since they have no bearing on DNI. Surface pressure, total column ozone and Ångstrom’s exponent are fixed to the long-term mean values observed at each location, calculated from daily observations. Since the impact of precipitable water on DNI is higher than the impact of the latter three variables, a random process similar to the case of the Ångstrom turbidity parameter is used to generate 3 × 10^4^ precipitable water samples. In this process, possible correlations between Ångström turbidity parameter and precipitable water are accounted for by separately modelling the frequency distribution of precipitable water for multiple ranges of the Ångström turbidity parameter. Nonetheless, in contrast to the Ångström turbidity parameter, the precipitable water samples are always drawn from the daily data distribution. Thus, only the effect of the AOD time scale on DNI is considered in the calculations. The solar zenith angle values are drawn randomly from their frequency distribution obtained for a 10-min temporal resolution at each site.

It must be here stressed that the evaluation of the impact of AOD’s time scale on the calculated long-term DNI does not require an all-sky solar radiation model, which would also take cloud extinction into consideration. This is because, first, the direct radiative effect of aerosols is only significant under clear-sky conditions. (Under cloudy conditions, cloud extinction overwhelms any other source of extinction.) Second, DNI is modelled as the product of individual transmittances, each corresponding to a specific atmospheric constituent (including, particularly, clouds and aerosols separately). Therefore, because (i) the variation of the long-term DNI as a function of AOD’s time scale is here evaluated as a ratio with respect to the long-term DNI obtained with daily AOD, and (ii) only the aerosol transmittance is varied, all the individual transmittance factors other than aerosol transmittance cancel out, including particularly the direct transmittance of clouds. Hence, using only a clear-sky solar radiation model is perfectly valid here.

### Global analysis

To obtain a spatially-continuous estimate of the long-term DNI variation induced by the AOD representation time scale, the methodology explained above has been applied pixel-wise to the worldwide daily gridded estimates of AOD for the period 2002α2014 produced by NASA’s Modern Era Retrospective Analysis for Research and Applications Aerosol Reanalysis (MERRAero)^35^,^38^. MERRAero is a parallel reanalysis of the NASA Modern Era Retrospective Analysis for Research (MERRA)^51^ produced using the Goddard Earth Observing System version 5 (GEOS-5)^52^ coupled with an aerosol transport module based on the Goddard Chemistry, Aerosol, Radiation, and Transport Model (GOCART)^53^. In addition to providing data assimilation of traditional meteorological parameters, the system includes assimilation of bias-corrected AOD observations from the Moderate Resolution Imaging Spectroradiometer (MODIS) sensor on both the Terra and Aqua satellites. The AOD assimilation algorithm involves cloud screening, homogenization and bias correction by means of a neural network scheme. The simulation is performed at a horizontal resolution of 0.5 degree latitude by 0.625 degree longitude, with 72 vertical layers extending up to 0.01 hPa (~80 km) and 3-hourly estimates^35^.

The coarse spatial resolution of MERRAero may raise concerns about its representativeness at local scales. A recent study^54^ has evaluated the impact of the different spatial samplings in the agreement between model estimates and ground observations. It concludes that the differences entirely due to spatial sampling mismatches are often even larger than the real measurement errors. However, the authors also state that temporal averaging over a month of data considerably reduces these differences, noting a threefold reduction in the particular case of AOD. In our study, we are dealing with long-term estimates, spanning multi-year periods. Then, it is expected that the impact of the coarse resolution of the MERRAero dataset at local scales is significantly alleviated. A detailed evaluation of these mismatches is out of the scope of this study.

In summary, the long-term DNI resource is computed from the MERRAero’s AOD using the REST2 clear-sky model, separately using AOD time scales from daily to monthly in 1-day time steps, as discussed above. The bias is then evaluated in each case against the long-term DNI obtained from the modelled daily AODs, used as skill reference.

## Results

**Figure 2:**
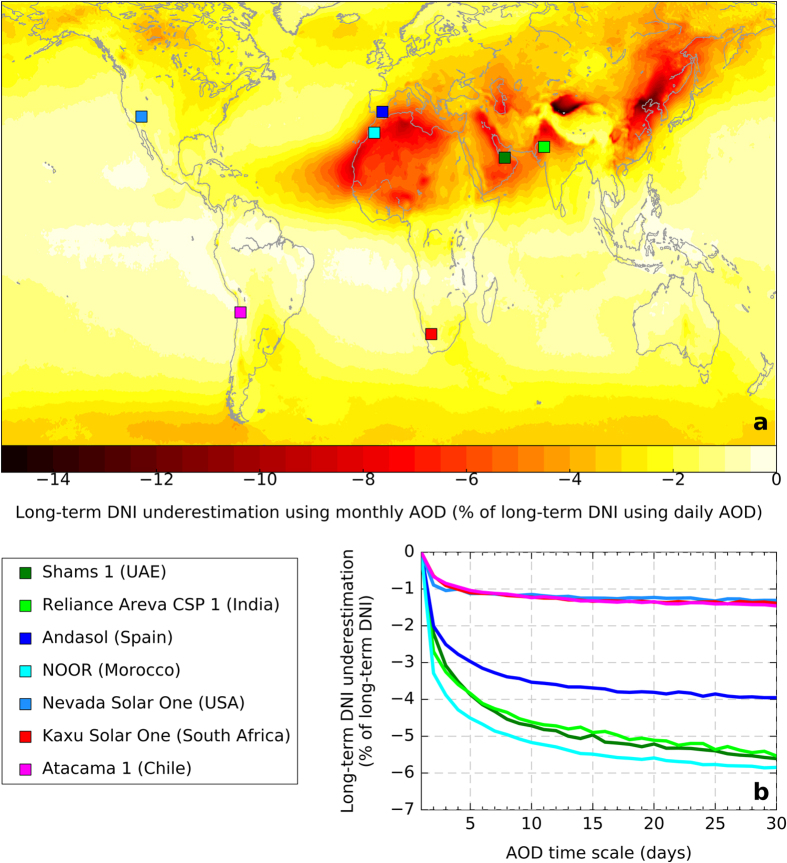
Relative bias in the computed long-term DNI using AOD at multiple time scales: (**a**) Using 30-day (monthly) AOD values; (**b**) Using AOD time scales from 1 to 30 days at the seven CSP actual plant locations shown on the map in the left panel (with same colour codes). (Map is generated with the Matplotlib version 1.5.1 and Basemap version 1.0.8 Python libraries [URL: http://matplotlib.org/ and http://matplotlib.org/basemap/]).

Overall, the bias describing the difference between the DNI resource calculated from multi-day average AOD data as opposed to daily AOD data is always found negative (i.e., the long-term DNI obtained from daily AODs is always greater than the case with coarser AODs). Figure 2a shows, in particular, the long-term DNI underestimation resulting from the use of monthly MERRAero AOD values. The spatial pattern of this underestimation resembles the spatial patterns of the worldwide long-term mean and standard deviation of AOD (see Supplementary Fig. S2). Specifically, greater underestimations often occur where AOD is higher and more variable. In absolute value, the calculated underestimations reach more than 2% in ≈42% of the land areas and more than 5% in ≈8% of the land areas. Remarkably, the latter apparently small fraction of land area coincides with some of the regions with the highest solar resource.

For instance, the location of seven existing or just commissioned large CSP plants is overlaid for reference on the DNI bias map shown in Fig. 2a. These sites are specifically considered here because they represent very diverse geographical areas, and have presumably been selected for their high DNI resource. For each location, the expected site-specific long-term DNI underestimation is shown in Fig. 2b as a function of the AOD time scale. At four of the sites (Andasol, Reliance Areva CSP 1, Shams 1 and NOOR), underestimations of the calculated long-term DNI greater than 4% (in absolute value) can be expected if DNI is evaluated using a monthly AOD time frame. The expected underestimation even reaches −6% in the case of NOOR, in Morocco. Note that some of these biases are similar or greater than the 5% bias considered earlier for a reference CSP plant in southern Spain, whose ideal characteristics are represented here by the case of the actual Andasol CSP plant. Three of these sites (Reliance Areva CSP 1, Shams 1 and NOOR) are located in areas with high prevalence of coarse aerosol particles, as indicated by the small mean Ångström’s exponent measured at the nearest AERONET sites (0.60 at the Karachi station, 0.73 at the Masdar Institute station and 0.53 at the Ouarzazate station, respectively) and relatively high mean AOD values (0.50, 0.39 and 0.15, respectively). At the Andasol site, which is only occasionally affected by Saharan dust outbreaks, the mean Ångström exponent measured at the nearest AERONET station (Granada) is 1.10 and the mean AOD is 0.15.

**Figure 3:**
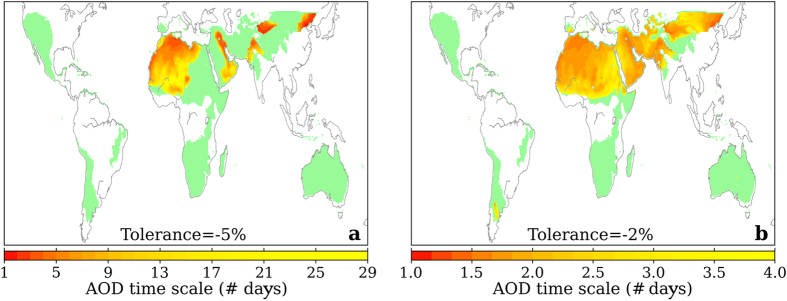
Land areas with long-term DNI greater than 2000 kWh/m^2^ according to the SSE database (coloured areas), and required AOD time scale to keep the bias due to the temporal representation of AOD within a tolerance level of −5% (**a**) and −2% (**b**) (yellowish and reddish areas). (Map is generated with the Matplotlib version 1.5.1 and Basemap version 1.0.8 Python libraries [URL: http://matplotlib.org/ and http://matplotlib.org/basemap/]).

As shown in Fig. 2b, the expected negative bias of the calculated long-term DNI rapidly increases (in absolute value) when the AOD representation time scale gets longer than one day, then reaches an asymptotic saturation level. Therefore, for any location, the AOD representation time scale required to keep the AOD-induced long-term DNI bias within some predefined tolerance level can be determined. Figure 3 shows the case for two tolerance values, −5% and −2%. In order to focus on the areas with high solar resource, only those areas with long-term DNI greater than a reference value of 2000 kWh/m^2^ are considered from the calculated value in the NASA Surface meteorology and Solar Energy (SSE) Release 6.0 dataset^55^,^56^.

The high-resource areas, which are highlighted in various colours in Fig. 3a,b, spread over six continents including areas of the North and South Americas, Africa, Europe, Asia and Australia, thus demonstrating the high coverage potential of concentrating solar technologies (the entire worldwide long-term DNI map from the SSE dataset is rather shown in Supplementary Fig. S3). When a bias of up to −5% is tolerated, only the yellowish and reddish land areas in Fig. 3a would require the use of AOD time scales finer than 30 days. These areas are prevalent over the Western and Central Sahara region, United Arab Emirates, Western Persian Gulf and Asian areas in the Hexi Corridor (southern Himalayas), Taklamakan desert (northern Himalayas) and Eastern China (around Beijing). Interestingly, some of these regions even require shorter AOD time scales of a few days only. For the rest of areas (highlighted in green colour) the use of monthly AODs would result in contributions to the overall estimation bias of long-term DNI lower than 5%, in absolute terms. However, requiring a tolerance level of only −2% seems more realistic from an application point of view, e.g. to satisfy more stringent bankability requirements.

This is the case shown in Fig. 3b. In contrast to Fig. 3a, the required AOD time scales now shorten drastically. The Sahara and Sahel regions, Middle East, and an important part of the high-resource areas of Asia require AOD time scales shorter than 4 days, and even shorter than 2 days for many of them. Nevertheless, very interestingly, these results also demonstrate that there are other important regions for concentrating solar technologies where long-term DNI can be safely evaluated (at least, to a tolerance level of −2%) using the most common approach of monthly AODs. These regions include Australia, South Africa, South and Central Americas and the Western United States.

**Figure 4:**
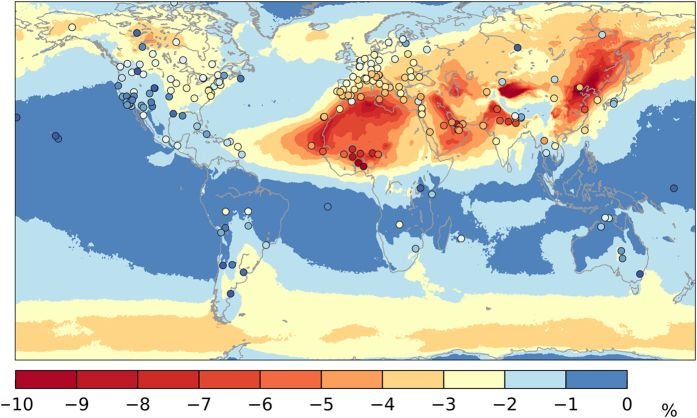
Differences of the long-term DNI calculated using monthly and daily AODs, in relative terms with respect to the value obtained from daily AODs. The map’s background represents the differences using AOD from MERRAero. The overlaid circles represent the differences obtained using AOD data from 214 AERONET test stations (the complete list is in Supplementary Table S1). (Map is generated with the Matplotlib version 1.5.1 and Basemap version 1.0.8 Python libraries [URL: http://matplotlib.org/ and http://matplotlib.org/basemap/]).

All these results are entirely based on MERRAero AOD data. As it is shown in Fig. 4, these results agree reasonably well with those derived from AERONET observations, particularly over areas of interest for concentrating solar technologies, as outlined in Fig. 3. In contrast, Fig. 4 also suggests that MERRAero’s representation of AOD at time scales from daily to monthly might correspond poorly with AERONET observations over a few regions, such as the Amazon rainforest or high-latitude areas of the Northern Hemisphere (North America and Europe). This could be caused by cloud artifacts. However, since cloudiness significantly reduces the solar resource, the impact of the problem is limited in the present context because the solar resource is, in any case, too low for CSP applications in these regions.

## Discussion

In the current context of advanced concentrating solar applications that require unbiased determinations of the long term DNI resource, long time series of accurate AOD data are required as inputs to the radiation models to provide DNI at the desirable high spatio-temporal resolution. To that end, the provision of reliable and unbiased worldwide daily AOD estimates from satellite platforms is still limited. The emerging capabilities of chemical transport models of the atmosphere open up new avenues to tackle this issue. A representative example that is used here to generalize results globally is the MERRAero model of NASA.

Using daily AOD data from both site-specific observations from a limited set of 214 AERONET ground stations and gridded model estimates from the MERRAero reanalysis for the period 2002–2014, a methodology has been developed to evaluate how the calculated long-term DNI resource is affected by using AOD data averaged over periods from 1 to 30 days. This methodology is based on moving averages and clear-sky DNI calculations using a large number of AOD conditions statistically selected from typical AOD frequency distributions in a Monte Carlo technique.

It is found that, among all geographic areas where the development of concentrating solar technologies is feasible, the magnitude of the modelled DNI solar resource can be significantly impacted (up to about 10%) by the temporal resolution of the underlying AOD data. The affected regions match those where the mean annual AOD and its variability (standard deviation) are both high, in which case a negative bias in the predicted mean annual DNI of up to 10% can be expected if mean monthly AOD data are used to evaluate it. Consequently, it is recommended that over regions where high-AOD conditions prevail, such as northern Africa, the Middle East or Asia, daily AOD be used to estimate the DNI resource. Conversely, other high-DNI but low-AOD areas, such as most of the Americas, southern Africa or Australia, should not be remarkably affected by the use of monthly-mean AOD data as input to solar radiation models. Regions where the induced negative bias in the predicted DNI is larger than 2 to 5% are at risk of underestimating the solar resource, and thus the annual revenue expected from large concentrating solar plants, which can affect the bankability of proposed projects at the financing stage.

Whereas the temporal averaging effect studied here always results in an underestimated solar resource, it must be stressed that any significant bias in the assumed AOD (in this case study, the MERRAero’s AOD) may overlap, ultimately resulting in either underestimation or overestimation of the solar resource. This specific issue is beyond the scope of this paper, since it is addressed in other publications. This risk of bias requires attention because it has been identified as a serious source of concern in previous studies^9^,^19^,^57^. Regions that are prone to this issue have been identified here by comparing the magnitude of gridded AOD data from MERRAero to reference ground observations from AERONET, which can be assumed free of bias (See Supplementary Fig. S2).

The results shown in Figs 3 and 4 (also in Supplementary Fig. S3) are relevant to Europe in the context of the conceptual idea of supplying power from CSP plants installed in the Middle-East and North Africa desert regions—idea that was supported until recently by the Desertec^58^ and Dii^59^ foundations. Based on the results shown in Fig. 3a,b, the long-term DNI potential, and thus the CSP potential, of these areas would be better evaluated from daily AOD data.

Finally, this study sets out a reference framework to delineate areas where improvements in remote-sensed AOD data and in numerical modelling of the atmospheric transport of aerosols would be more urgent in the context of intensive solar power applications. Using various avenues, work is underway by these authors and many world institutions to provide better aerosol data in terms of both absolute accuracy and higher spatio-temporal resolution, with the goal of deriving more accurate modelled DNI databases, thus serving the growing needs of the solar industry.

## Additional Information

**How to cite this article**: Ruiz-Arias, J. A. *et al*. Worldwide impact of aerosol’s time scale on the predicted long-term concentrating solar power potential. *Sci. Rep.* **6**, 30546; doi: 10.1038/srep30546 (2016).

## Supplementary Material

Supplementary Information

## Figures and Tables

**Figure 1:**
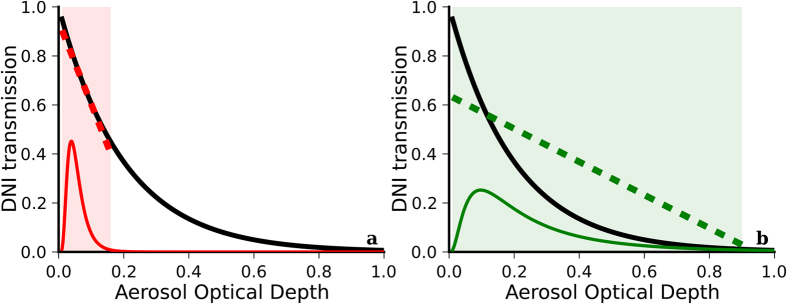
(**a**) Schematic representation of the direct transmittance, *T*_*a*_(*τ*), through an aerosol layer (black solid curve) compared to the typical AOD data distribution (red solid curve) at a low-turbidity site. (**b**) AOD data distribution (green solid curve) at a high-turbidity site. The dashed lines represent hypothetical linear approximations of the DNI aerosol transmittance. The shaded areas highlight the range of AOD values at each site. The Y-axis scale for the AOD data distribution is not shown (arbitrary unit, not relevant).
