# Supplementary material for: Worldwide impact of aerosol’s time scale on the predicted long-term concentrating solar power potential
Source: Sci Rep. 2016 Aug 10;6:30546. doi: 10.1038/srep30546 (PMC4979049; doi:10.1038/srep30546)
Supplement: Supplementary Information [file srep30546-s1.pdf]

Worldwide impact of aerosol's time scale on the predicted long-term concentrating solar power potential

Jose A. Ruiz-Arias[1,2,*], Christian A. Gueymard[3], Francisco J. Santos-Alamillos[2], David Pozo-Vázquez[2]

[1] Department of Applied Physics I, University of Málaga, Málaga, Spain

[2] Solar Radiation and Atmosphere Modeling Group, Department of Physics, University of Jaén, Jaén, Spain

[3] Solar Consulting Services, Colebrook, NH, USA

Corresponding Author: José A Ruiz-Arias, Department of Applied Physics I, Sciences Faculty, University of Málaga, 29071, Málaga, Spain. E-mail: jararias@ujaen.es

Supplementary Figure S1: Variation of the AOD frequency distribution characteristics as a function of AOD time scale for an AERONET site with low-turbidity conditions (Goddard Space Flight Center, GSFC), and an AERONET site with high-turbidity conditions (Capo Verde). (a) Variation of AOD frequency distribution's shape at GSFC. (b) Variation of AOD frequency distribution's shape at Capo Verde. (c) Relative variation of mean and standard deviation values at GSFC. (d) Relative variation of mean and standard deviation values at Capo Verde. The relative variations refer to the daily time scale. (Maps are generated with the Matplotlib version 1.5.1 and Basemap version 1.0.8 Python libraries [URL: http://matplotlib.org/ and http://matplotlib.org/basemap/]).

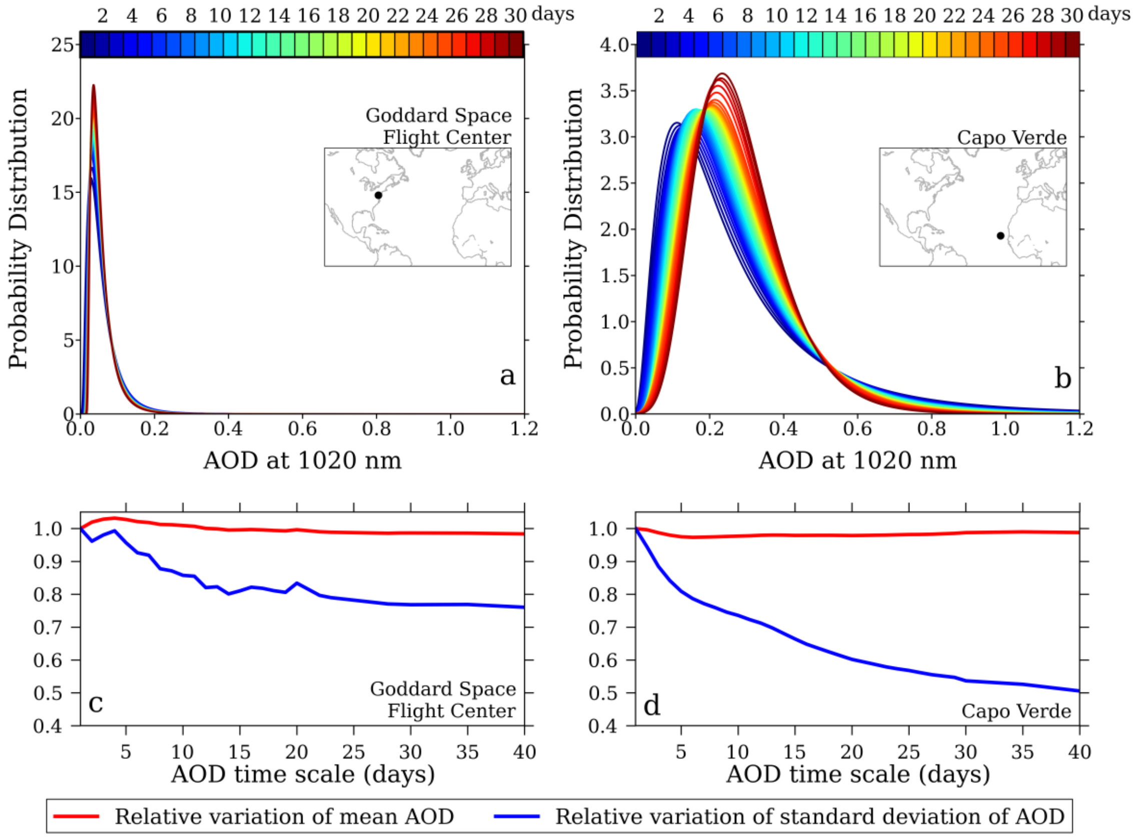

The frequency distributions of AOD shown at different time scales in this figure are computed using a moving average process with an averaging window of varying size, from daily to monthly, in progressive 1-day steps. This process is repeated for all available AOD observations at 214 sites of the Aerosol Robotic Network (AERONET). This worldwide network offers high-quality AOD observations[48] that are widely considered the ultimate reference to validate other sources of AOD data. Overall, the AOD mean value (red curves in Figs. S1c and S1d) remains approximately constant over time scales from daily to monthly. The standard deviation (blue curves in Figs. S1c and S1d), however, decays monotonically. At monthly scales for some sites, this decay even reaches 60% of the standard deviation at daily scale

Supplementary Figure S2 Ångström's aerosol turbidity coefficient (i.e., aerosol optical depth at 1 µm) from the MERRAero dataset (map background) and AERONET stations (circles). (a) Long-term mean value, and (b) long-term standard deviation. (Maps are generated with the Matplotlib version 1.5.1 and Basemap version 1.0.8 Python libraries [URL: http://matplotlib.org/ and http://matplotlib.org/basemap/]).

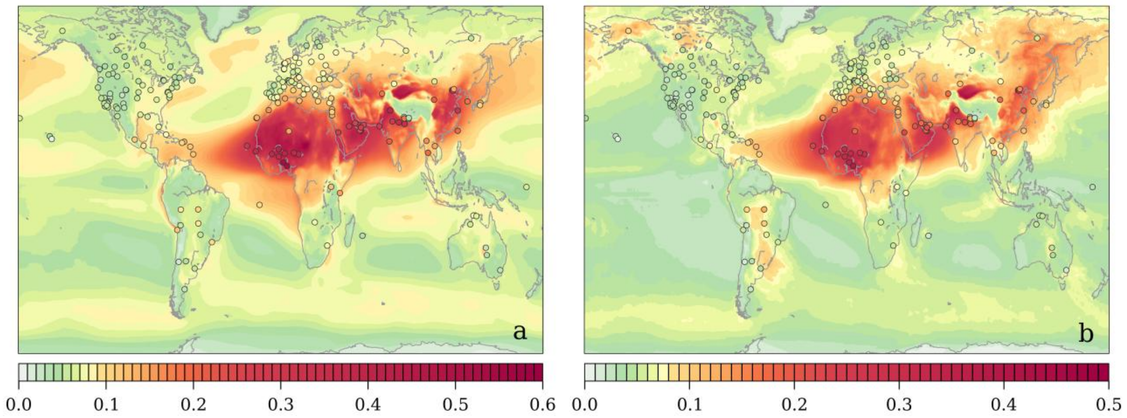

Both the long-term mean and standard deviation values of the Ångström's aerosol turbidity coefficient from the MERRAero dataset (see Methods section) agree reasonably well with the long-term values evaluated at 214 AERONET sites worldwide. The major disagreements appear over the Amazon rainforest region.

In the regions with higher standard deviations (Fig. S2b), the change in long-term DNI with AOD's temporal scale is greater. These regions also coincide with those with greater mean aerosol turbidity coefficient (compare with Fig. S2a).

Supplementary Figure S3: Long-term annual DNI for the world. (a) Estimate from NASA's Surface meteorology and Solar Energy (SSE) database (see comments below). (b) Calculated long-term DNI bias that would affect the resource data in (a) if monthly rather than daily aerosol optical depth data were used to produce (a). (Maps are generated with the Matplotlib version 1.5.1 and Basemap version 1.0.8 Python libraries [URL: http://matplotlib.org/ and http://matplotlib.org/basemap/]).

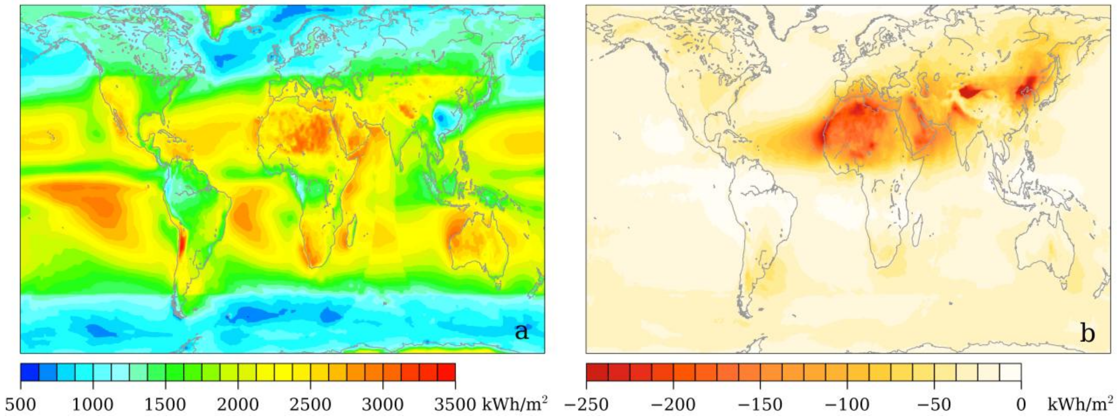

The SSE dataset accounts for the direct effect of aerosols from a monthly climatology obtained from six years of results from the MATCH aerosol transport model. The monthly mode of their AOD predictions is actually used instead of the monthly mean to mitigate the bias issue that is in turn quantified globally in this study[56]. Further details are available at https://eosweb.larc.nasa.gov/sse/documents/SSE6Methodology.pdf. Note that SSE is probably not the most accurate DNI dataset, considering its coarse spatial resolution (1x1°) and other factors. However, it is in the public domain and its entire methodology is known, contrarily to other newer and more specialized—but commercial—datasets. Thus, SSE is a good target dataset for the present research purposes. We would like to acknowledge these data were obtained from the NASA Langley Research Center Atmospheric Science Data Center Surface meteorological and Solar Energy (SSE) web portal supported by the NASA LaRC POWER Project.

Supplementary Table S1 (and companion discussion): Inventory of the Aerosol Robotic Network (AERONET) test sites used in this study and ancillary information. Further information on the AERONET dataset and the data used here is provided in the Methods section.

The information provided in the columns is as follows: *Station* is the AERONET station name, *Latit.* is the latitude in degrees north, *Longit.* is the longitude in degrees east. The columns $E[\alpha_i]$ and $E[\tau_i]$ are the long-term mean Ångström's exponent and Ångström's turbidity parameter (i.e., AOD at 1 μm) obtained from instantaneous observations, respectively. The Ångström's turbidity parameter is calculated from multi-spectral instantaneous AOD observations between 440 and 870 nm using the Ångström's empirical formula $\tau = \beta\lambda^{-\alpha}$, where β is the Ångström's turbidity parameter, α is the Ångström's exponent, and τ is an observed AOD at a wavelength λ (in microns) between 440 and 870 nm. Both, $E[\alpha_i]$ and $E[\tau_i]$ describe the aerosol climatology of each test site.

From the time series of instantaneous observations of β at every site, the departures, ε_β, from each daily mean are accumulated over the entire record period and used to compute the long-term standard deviation of β at sub-daily scale (column $SD[\tau_{i,d}]$) as the square root of $E[\varepsilon_\beta^2]$, where $E[\cdot]$ represent the average value. It accounts for the sub-daily variability of AOD.

From the frequency distribution of instantaneous observations of β, $P(\beta)$, the long-term mean DNI, $E[DNI_d]$, is computed at every station as $\int_0^\infty P(\beta)B(\beta)d\beta$, where $B(\beta)$ is the DNI value computed using the REST2 clear-sky model (described in the Methods section) with the following fixed input parameter values: 1.5 for optical air mass, 1013.25 mb for surface pressure, 330 DU for total column amount of ozone, 0.92 for aerosol single-scattering albedo, 0.70 for aerosol asymmetry parameter, and precipitable water content and Ångström's exponent fixed to the long-term averages observed at each AERONET station.

In contrast to this approach, which accounts for the sub-daily variations of AOD, the long-term mean DNI can be estimated with REST2 from the distribution of daily β observations. This approach neglects the sub-daily variations of AOD. The differences between this latter value and the former one are shown in the column $\Delta[DNI_d]$ for each station. At 203 out of the 214 test stations, the difference is less than 2%, which roughly corresponds to a mean deviation of about 7 W m^{-2}. This deviation is smaller than expected deviations in many DNI solar radiation models or even in some ground observations. Hence, as assumed in this study, a daily representation of AOD appears sufficient and commensurate with the current requirements and state-of-the-art in solar energy applications.

The column $E[\tau_d]$ shows the long-term mean Ångström turbidity parameter obtained from the observed daily means. Note the very small deviation with respect to the long-term mean value obtained from instantaneous observations (i.e., $E[\tau_i]$), as it is already shown for two AERONET stations in Supplementary Fig. S1c-d.

From the time series of daily mean β, the departures from each monthly mean are accumulated over the entire record period at every station and used to compute the long-term standard deviation of daily β at sub-monthly scale (column $SD[\tau_{d,m}]$) as for the sub-daily case described above. This parameter accounts for the sub-monthly variability of daily β. Note this is a similar procedure as the one followed to compute $SD[\tau_{i,d}]$. The comparison of $SD[\tau_{d,m}]$ and $SD[\tau_{i,d}]$ shows that the sub-daily variability of AOD is much lower, in average, than the daily variability

at sub-monthly scale. (At many sites the daily variability is up to two orders of magnitude larger than the sub-daily variability). This result is aligned with one of the major assumptions made in this study, in virtue of which the AOD variability at sub-daily scale is neglected with respect to the variability at synoptic scales.

As for sub-daily scale, but at monthly scale, the columns $E[DNI_m]$ and $\Delta[DNI_m]$ compare the calculation of the long-term mean DNI considering the day-by-day variation of β and approximating this value as simply the calculated DNI from the monthly distribution of β observations. In this case, as expected consistently with the results presented in this study, the differences at assuming a monthly β with respect to the more correct day-by-day calculation are much larger, being greater or equal than 3% at 24 of the test sites (highlighted in bold). Typically, these sites are characterized by high mean β and low mean α (i.e., coarse particles, such as desert dust).

Note that an important difference of this latter result with respect to the more comprehensive evaluation carried out using the Monte-Carlo analysis is that here a fixed optical air mass of 1.5 (i.e., fixed solar position) is assumed, which is not necessary in the stochastic framework. Note also that $E[DNI_d]$ is lower than $E[DNI_m]$. The rationale behind is that the latter is a calculation based on monthly periods while the former is based on a much longer multi-year period (indeed, the entire record period in each site).

Station	Latit.	Longit.	$E[\alpha_i]$	$E[\tau_i]$	$SD[\tau_{i,d}]$	$E[DNI_d]$	$\Delta[DNI_d]$	$E[\tau_d]$	$SD[\tau_{d,m}]$	$E[DNI_m]$	$\Delta[DNI_m]$
ATHENS-NOA	37.988	23.775	1.37	0.08	0.001	800	0.60%	0.08	0.084	809	-1.00%
**Abu_Al_Bukhoosh**	**25.495**	**53.146**	**0.67**	**0.29**	**0.006**	**589**	**0.10%**	**0.28**	**0.228**	**621**	**-4.60%**
**Agoufou**	**15.345**	**-1.479**	**0.29**	**0.42**	**0.010**	**512**	**-0.60%**	**0.45**	**0.373**	**529**	**-7.70%**
Alta_Floresta	-9.871	-56.104	1.36	0.11	0.003	745	1.40%	0.13	0.165	732	-1.50%
Ames	42.021	-93.775	1.34	0.06	0.001	835	1.30%	0.06	0.048	849	-0.50%
Arcachon	44.664	-1.163	1.21	0.06	0.000	846	0.60%	0.06	0.047	847	-0.50%
Arica	-18.472	-70.313	1.05	0.12	0.001	761	0.00%	0.12	0.063	760	-0.60%
Ascension_Island	-7.976	-14.415	0.66	0.10	0.000	818	0.00%	0.10	0.050	819	-0.30%
Autilla	41.997	-4.603	1.24	0.04	0.000	880	0.90%	0.04	0.042	887	-0.30%
Avignon	43.933	4.878	1.44	0.06	0.001	821	0.70%	0.07	0.056	830	-0.80%
BONDVILLE	40.053	-88.372	1.43	0.06	0.001	818	1.40%	0.07	0.068	825	-0.60%
BSRN_BAO_Boulder	40.045	-105.006	1.30	0.04	0.000	886	0.40%	0.04	0.027	892	-0.10%
**Bahrain**	**26.208**	**50.609**	**0.70**	**0.24**	**0.005**	**629**	**0.20%**	**0.25**	**0.184**	**647**	**-3.00%**
**Banizoumbou**	**13.541**	**2.665**	**0.36**	**0.40**	**0.011**	**521**	**-0.30%**	**0.43**	**0.383**	**535**	**-7.40%**
Barcelona	41.386	2.117	1.37	0.07	0.001	819	0.90%	0.07	0.058	829	-0.70%
**Beijing**	**39.977**	**116.381**	**1.09**	**0.26**	**0.013**	**572**	**0.60%**	**0.28**	**0.297**	**602**	**-8.70%**
Belsk	51.837	20.792	1.47	0.07	0.001	800	0.50%	0.08	0.056	804	-0.70%
Billerica	42.528	-71.269	1.47	0.04	0.000	860	1.50%	0.04	0.039	874	-0.30%
Birdsville	-25.899	139.346	0.89	0.03	0.001	887	3.30%	0.03	0.050	914	0.00%
Blida	36.508	2.881	0.95	0.13	0.002	740	0.60%	0.14	0.137	755	-1.90%
Bonanza_Creek	64.743	-148.316	1.33	0.06	0.002	823	3.40%	0.06	0.100	848	0.00%
Bozeman	45.662	-111.045	1.48	0.03	0.000	887	1.00%	0.03	0.030	892	-0.10%
Bratts_Lake	50.280	-104.700	1.47	0.04	0.000	871	1.00%	0.04	0.034	881	-0.20%
Bucharest_Inoe	44.348	26.030	1.56	0.08	0.001	777	0.20%	0.09	0.065	785	-1.30%

Supplementary Table S1: Continued

Station	Latit.	Longit.	E[α_i]	E[τ_i]	SD[$\tau_{i,d}$]	E[DNI_d]	Δ[DNI_d]	E[τ_d]	SD[$\tau_{d,m}$]	E[DNI_m]	Δ[DNI_m]
Burjassot	39.508	-0.418	1.21	0.06	0.001	833	0.90%	0.07	0.063	837	-0.70%
CARTEL	45.379	-71.931	1.44	0.04	0.000	857	1.10%	0.05	0.041	867	-0.50%
CASLEO	-31.799	-69.306	0.97	0.01	0.000	933	1.00%	0.01	0.014	942	0.00%
CCNY	40.821	-73.949	1.48	0.05	0.000	841	0.80%	0.05	0.050	847	-0.50%
CEILAP-BA	-34.567	-58.500	1.09	0.05	0.000	873	0.60%	0.05	0.035	879	-0.20%
COVE	36.900	-75.710	1.65	0.07	0.001	802	1.10%	0.07	0.066	813	-0.70%
COVE_SEAPRISM	36.900	-75.710	1.57	0.04	0.000	877	0.30%	0.04	0.037	874	-0.30%
CRPSM_Malindi	-2.996	40.194	0.57	0.14	0.001	762	0.10%	0.15	0.080	761	-0.70%
CUIABA-MIRANDA	-15.729	-56.021	1.34	0.09	0.001	779	0.50%	0.10	0.123	779	-0.50%
CUT-TEPAK	34.675	33.043	1.23	0.09	0.001	780	1.10%	0.10	0.131	796	-1.70%
Cabauw	51.971	4.927	1.23	0.09	0.001	786	0.30%	0.09	0.064	792	-0.90%
Cabo_da_Roca	38.783	-9.500	1.00	0.06	0.001	842	0.80%	0.07	0.079	850	-0.60%
Caceres	39.479	-6.343	1.38	0.05	0.000	855	1.20%	0.05	0.058	863	-0.60%
CalTech	34.137	-118.126	1.13	0.06	0.000	854	0.50%	0.06	0.040	852	-0.30%
Camaguey	21.422	-77.850	0.84	0.08	0.000	829	0.40%	0.08	0.058	835	-0.20%
Campo_Grande_SONDA	-20.438	-54.538	1.30	0.05	0.000	851	0.50%	0.06	0.078	849	0.00%
Canberra	-35.271	149.111	1.20	0.02	0.000	911	0.70%	0.03	0.017	917	-0.10%
Cape_San_Juan	18.384	-65.620	0.45	0.12	0.001	794	0.30%	0.11	0.116	807	-0.50%
**Capo_Verde**	**16.733**	**-22.935**	**0.30**	**0.29**	**0.004**	**620**	**0.10%**	**0.29**	**0.246**	**644**	**-3.70%**
Carpentras	44.083	5.058	1.46	0.06	0.000	837	0.70%	0.06	0.054	846	-0.70%
Cart_Site	36.607	-97.486	1.31	0.05	0.000	853	0.50%	0.05	0.037	856	-0.20%
Chapais	49.822	-74.975	1.55	0.04	0.000	872	1.20%	0.04	0.034	884	-0.30%
Chen-Kung_Univ	23.000	120.217	1.33	0.20	0.004	620	-0.60%	0.20	0.127	631	-2.50%
Chiang_Mai_Met_Sta	18.771	98.972	1.40	0.17	0.001	647	-0.30%	0.19	0.139	642	-2.60%
Churchill	58.736	-93.818	1.59	0.04	0.001	857	1.80%	0.04	0.046	875	-0.20%
Cordoba-CETT	-31.524	-64.464	1.18	0.04	0.000	877	0.80%	0.04	0.034	883	-0.20%
**DMN_Maine_Soroa**	**13.217**	**12.023**	**0.39**	**0.38**	**0.016**	**534**	**0.10%**	**0.41**	**0.360**	**544**	**-6.70%**
**Dakar**	**14.394**	**-16.959**	**0.36**	**0.37**	**0.006**	**545**	**-0.20%**	**0.39**	**0.303**	**562**	**-5.30%**
Dalanzadgad	43.577	104.419	1.12	0.06	0.001	839	2.10%	0.06	0.086	856	-0.50%
Darwin	-12.424	130.892	1.21	0.07	0.001	821	0.70%	0.08	0.048	819	-0.30%
Davos	46.813	9.844	1.39	0.03	0.000	889	1.30%	0.03	0.033	900	-0.10%
**Dhabi**	**24.481**	**54.383**	**0.61**	**0.29**	**0.004**	**592**	**0.00%**	**0.29**	**0.228**	**609**	**-3.60%**
Dhadnah	25.513	56.325	0.72	0.25	0.002	623	-0.20%	0.25	0.172	637	-2.10%
**Djougou**	**9.760**	**1.599**	**0.52**	**0.54**	**0.013**	**414**	**-1.30%**	**0.53**	**0.369**	**455**	**-9.50%**
Dry_Tortugas	24.628	-82.872	1.14	0.07	0.001	821	0.50%	0.08	0.065	824	-0.60%
Dunkerque	51.035	2.368	1.20	0.08	0.001	804	0.60%	0.08	0.051	815	-0.60%
**Dushanbe**	**38.553**	**68.858**	**0.73**	**0.18**	**0.003**	**695**	**0.60%**	**0.18**	**0.202**	**725**	**-3.10%**
EVK2-CNR	27.959	86.813	0.86	0.02	0.000	919	1.50%	0.02	0.020	930	0.00%
Egbert	44.226	-79.750	1.55	0.04	0.000	859	0.90%	0.05	0.041	863	-0.50%
Eilat	29.503	34.917	0.85	0.13	0.002	756	1.00%	0.13	0.150	768	-1.80%
El_Arenosillo	37.105	-6.733	1.09	0.08	0.001	817	0.80%	0.08	0.085	829	-0.90%
Ersa	43.004	9.359	1.35	0.06	0.000	837	0.80%	0.06	0.060	847	-0.90%
Evora	38.568	-7.912	1.21	0.06	0.001	839	1.40%	0.06	0.067	856	-0.60%
FORTH_CRETE	35.333	25.282	1.15	0.09	0.001	783	0.90%	0.10	0.089	798	-1.30%

Supplementary Table S1: Continued

Station	Latit.	Longit.	E[α_i]	E[τ_i]	SD[$\tau_{i,d}$]	E[DNI_d]	Δ[DNI_d]	E[τ_d]	SD[$\tau_{d,m}$]	E[DNI_m]	Δ[DNI_m]
Fort_McMurray	56.752	-111.476	1.58	0.05	0.001	839	2.10%	0.04	0.049	865	-0.30%
Frenchman_Flat	36.809	-115.935	1.12	0.03	0.000	900	0.60%	0.03	0.022	907	-0.10%
Fresno	36.782	-119.773	1.17	0.06	0.000	841	0.40%	0.07	0.036	842	-0.30%
Fresno_2	36.785	-119.773	1.15	0.05	0.000	856	0.40%	0.06	0.031	858	-0.20%
GSFC	38.992	-76.840	1.57	0.06	0.001	826	0.90%	0.06	0.067	826	-0.60%
**Gandhi_College**	**25.871**	**84.128**	**0.98**	**0.34**	**0.004**	**506**	**-0.10%**	**0.34**	**0.175**	**525**	**-3.00%**
Gloria	44.600	29.360	1.55	0.05	0.000	839	0.30%	0.05	0.034	845	-0.60%
Goldstone	35.233	-116.792	0.93	0.03	0.000	910	0.80%	0.03	0.019	920	-0.10%
Granada	37.164	-3.605	1.04	0.08	0.001	806	0.90%	0.09	0.089	818	-0.90%
Guadeloup	16.333	-61.500	0.36	0.12	0.001	795	0.40%	0.13	0.126	799	-0.60%
Gustav_Dalen_Tower	58.594	17.467	1.42	0.03	0.000	887	0.60%	0.04	0.021	887	-0.20%
Gwangju_GIST	35.228	126.843	1.25	0.17	0.004	663	0.20%	0.18	0.132	675	-2.50%
HJAndrews	44.239	-122.224	1.49	0.03	0.000	894	1.20%	0.03	0.023	904	-0.10%
Hamburg	53.568	9.973	1.30	0.07	0.001	813	0.70%	0.07	0.051	824	-0.80%
Hamim	22.967	54.300	0.63	0.25	0.003	635	-0.10%	0.24	0.175	651	-2.00%
Harvard_Forest	42.532	-72.188	1.43	0.04	0.000	873	0.90%	0.04	0.034	881	-0.30%
Helsinki_Lighthouse	59.949	24.926	1.40	0.05	0.000	862	0.40%	0.05	0.033	867	-0.30%
Hermosillo	29.075	-110.960	1.02	0.05	0.000	872	0.30%	0.05	0.028	873	-0.10%
Howland	45.200	-68.733	1.62	0.04	0.000	863	1.50%	0.04	0.039	879	-0.40%
Huelva	37.016	-6.569	1.07	0.07	0.001	823	1.20%	0.07	0.080	834	-0.80%
ICIPE-Mbita	-0.417	34.200	1.03	0.11	0.001	765	0.40%	0.11	0.075	772	-0.80%
**IER_Cinzana**	**13.278**	**-5.934**	**0.35**	**0.38**	**0.009**	**540**	**-0.10%**	**0.39**	**0.328**	**563**	**-5.50%**
IMAA_Potenza	40.600	15.720	1.34	0.07	0.001	817	0.90%	0.06	0.064	839	-0.80%
IMC_Oristano	39.910	8.500	1.19	0.10	0.002	771	1.10%	0.10	0.136	791	-1.90%
IMS-METU-ERDEMLI	36.565	34.255	1.25	0.11	0.001	757	0.20%	0.11	0.099	759	-1.40%
ISDGM_CNR	45.437	12.332	1.58	0.10	0.001	735	0.60%	0.11	0.081	750	-2.10%
**Ilorin**	**8.320**	**4.340**	**0.61**	**0.54**	**0.009**	**398**	**-0.80%**	**0.51**	**0.412**	**460**	**-9.90%**
Ispra	45.803	8.627	1.54	0.09	0.001	760	0.80%	0.10	0.096	770	-2.00%
Issyk-Kul	42.623	76.983	1.21	0.07	0.001	821	1.10%	0.07	0.108	835	-0.60%
Izana	28.309	-16.499	0.95	0.04	0.000	869	2.10%	0.05	0.088	882	0.40%
Jabiru	-12.661	132.893	1.02	0.07	0.001	840	0.80%	0.07	0.051	837	-0.30%
Jaipur	26.906	75.806	0.75	0.28	0.004	592	-0.40%	0.28	0.172	600	-2.60%
Jomsom	28.778	83.714	1.55	0.04	0.000	861	1.90%	0.04	0.038	879	-0.10%
**KAUST_Campus**	**22.305**	**39.103**	**0.63**	**0.32**	**0.005**	**559**	**0.20%**	**0.34**	**0.333**	**586**	**-7.30%**
KONZA_EDC	39.102	-96.610	1.39	0.05	0.000	843	0.90%	0.05	0.041	852	-0.20%
Kanpur	26.513	80.232	0.91	0.35	0.006	504	-0.40%	0.35	0.232	523	-4.00%
Karachi	24.870	67.030	0.70	0.27	0.003	600	-0.10%	0.29	0.228	602	-2.90%
Kelowna	49.955	-119.373	1.48	0.04	0.000	866	1.00%	0.04	0.033	879	-0.20%
Kelowna_UAS	49.941	-119.400	1.45	0.04	0.000	875	1.20%	0.04	0.031	888	-0.10%
Kyiv	50.364	30.497	1.45	0.07	0.000	809	0.30%	0.07	0.047	815	-0.60%
La_Jolla	32.870	-117.250	1.20	0.05	0.000	865	0.60%	0.05	0.035	866	-0.20%
La_Laguna	28.482	-16.321	0.57	0.14	0.001	759	0.30%	0.13	0.163	789	-1.00%
La_Parguera	17.970	-67.045	0.62	0.10	0.001	806	0.40%	0.11	0.106	811	-0.60%
La_Paz	-16.539	-68.066	0.90	0.05	0.000	884	0.10%	0.05	0.022	884	-0.10%

Supplementary Table S1: Continued

Station	Latit.	Longit.	E[α_i]	E[τ_i]	SD[$\tau_{i,d}$]	E[DNI_d]	Δ[DNI_d]	E[τ_d]	SD[$\tau_{d,m}$]	E[DNI_m]	Δ[DNI_m]
Laegeren	47.480	8.351	1.50	0.06	0.000	835	0.70%	0.06	0.046	840	-0.70%
**Lahore**	**31.542**	**74.325**	**0.84**	**0.38**	**0.007**	**489**	**-0.60%**	**0.37**	**0.217**	**518**	**-4.60%**
Lake_Argyle	-16.108	128.749	1.06	0.06	0.001	854	1.10%	0.06	0.050	857	-0.20%
Lampedusa	35.517	12.632	0.95	0.11	0.001	772	0.60%	0.11	0.124	792	-1.70%
Lanai	20.735	-156.922	0.67	0.05	0.000	892	0.20%	0.05	0.022	894	-0.10%
Le_Fauga	43.384	1.285	1.50	0.07	0.001	816	0.50%	0.07	0.066	821	-1.00%
Lecce_University	40.335	18.111	1.36	0.08	0.001	788	0.90%	0.08	0.068	804	-1.20%
Leipzig	51.352	12.435	1.46	0.07	0.001	802	0.40%	0.08	0.056	809	-0.90%
Lille	50.612	3.142	1.34	0.08	0.001	798	0.70%	0.08	0.053	807	-0.80%
Mainz	49.999	8.300	1.43	0.07	0.001	803	0.50%	0.08	0.050	808	-0.80%
Malaga	36.715	-4.478	0.97	0.08	0.001	820	0.50%	0.08	0.088	823	-0.70%
Maricopa	33.069	-111.972	1.03	0.05	0.000	876	0.40%	0.05	0.028	881	-0.10%
MD_Science_Center	39.283	-76.617	1.62	0.06	0.000	825	0.90%	0.06	0.059	827	-0.60%
MVCO	41.300	-70.550	1.20	0.01	0.000	915	4.70%	0.01	0.006	957	0.00%
Messina	38.197	15.567	1.25	0.09	0.001	792	0.80%	0.09	0.079	806	-1.20%
Mexico_City	19.334	-99.182	1.49	0.11	0.002	746	-0.10%	0.11	0.063	747	-1.00%
Mezaira	23.145	53.779	0.68	0.25	0.004	630	0.20%	0.25	0.208	646	-2.50%
Mauna_Loa	19.539	-155.578	1.20	0.01	0.000	915	4.70%	0.01	0.006	957	0.00%
Midway_Island	28.210	-177.378	0.60	0.06	0.000	866	0.40%	0.06	0.043	868	-0.20%
Minsk	53.920	27.601	1.43	0.07	0.001	809	0.70%	0.07	0.063	818	-0.60%
Missoula	46.917	-114.083	1.46	0.04	0.000	858	1.30%	0.05	0.043	866	-0.20%
Modena	44.632	10.945	1.53	0.10	0.001	756	0.50%	0.10	0.078	765	-1.80%
Moldova	47.000	28.816	1.48	0.07	0.001	803	0.40%	0.07	0.050	808	-0.70%
Mongu	-15.254	23.151	1.71	0.08	0.000	774	0.00%	0.08	0.070	774	-0.90%
Monterey	36.593	-121.855	0.92	0.04	0.000	884	0.50%	0.05	0.034	885	-0.10%
Moscow_MSU_MO	55.700	37.510	1.50	0.07	0.001	800	0.50%	0.07	0.063	810	-0.60%
Mukdahan	16.607	104.676	1.36	0.16	0.001	675	-0.20%	0.16	0.104	677	-1.90%
Munich_University	48.148	11.573	1.47	0.06	0.000	819	0.80%	0.06	0.048	831	-0.80%
**Mussafa**	**24.372**	**54.467**	**0.69**	**0.27**	**0.004**	**605**	**0.20%**	**0.28**	**0.244**	**612**	**-3.50%**
Nauru	-0.521	166.916	0.45	0.06	0.000	883	0.10%	0.06	0.029	884	-0.10%
Nes_Ziona	31.922	34.789	1.01	0.13	0.003	738	1.00%	0.13	0.156	756	-2.10%
OHP_OBSERVATOIRE	43.935	5.710	1.46	0.04	0.000	865	1.20%	0.04	0.045	877	-0.40%
Oostende	51.225	2.925	1.31	0.08	0.001	805	0.50%	0.08	0.053	815	-0.60%
Osaka	34.651	135.591	1.36	0.12	0.002	732	0.70%	0.12	0.098	742	-1.40%
**Ouagadougou**	**12.200**	**-1.400**	**0.40**	**0.39**	**0.010**	**524**	**-0.30%**	**0.41**	**0.353**	**544**	**-6.60%**
Oujda	34.653	-1.899	1.46	0.04	0.000	865	1.20%	0.04	0.045	877	-0.40%
PEARL	80.054	-86.417	1.64	0.02	0.000	907	1.10%	0.02	0.018	914	-0.10%
Palaiseau	48.700	2.208	1.33	0.07	0.001	812	0.60%	0.07	0.048	819	-0.60%
Palencia	41.989	-4.516	1.36	0.05	0.001	856	1.40%	0.05	0.053	870	-0.50%
Paris	48.867	2.333	1.37	0.07	0.001	806	0.70%	0.08	0.049	814	-0.70%
Pickle_Lake	51.449	-90.218	1.52	0.03	0.000	877	1.20%	0.04	0.036	886	-0.30%
**Pokhara**	**28.151**	**83.971**	**1.35**	**0.18**	**0.001**	**650**	**-0.20%**	**0.19**	**0.150**	**650**	**-3.60%**
Pune	18.537	73.805	1.06	0.21	0.002	635	-0.30%	0.22	0.093	635	-1.50%
QOMS_CAS	28.365	86.948	1.37	0.07	0.001	806	0.70%	0.08	0.049	814	-0.70%

Supplementary Table S1: Continued

Station	Latit.	Longit.	$E[\alpha_i]$	$E[\tau_i]$	$SD[\tau_{i,d}]$	$E[DNI_d]$	$\Delta[DNI_d]$	$E[\tau_d]$	$SD[\tau_{d,m}]$	$E[DNI_m]$	$\Delta[DNI_m]$
REUNION_ST_DENIS	-20.883	55.483	0.66	0.04	0.000	903	0.20%	0.04	0.019	904	-0.10%
Ragged_Point	13.165	-59.432	0.29	0.12	0.001	801	0.10%	0.12	0.106	804	-0.60%
Railroad_Valley	38.504	-115.962	1.13	0.03	0.000	896	1.60%	0.03	0.026	910	-0.10%
Red_Mountain_Pass	37.908	-107.725	1.70	0.02	0.000	921	0.50%	0.02	0.015	927	0.00%
Resolute_Bay	74.733	-94.900	1.57	0.03	0.000	881	1.40%	0.03	0.030	899	-0.10%
Rimrock	46.487	-116.992	1.36	0.04	0.000	874	0.90%	0.04	0.038	880	-0.20%
Rio_Branco	-9.957	-67.869	1.56	0.09	0.001	768	0.60%	0.09	0.106	771	-1.00%
Rogers_Dry_Lake	34.926	-117.885	1.02	0.04	0.000	885	0.70%	0.04	0.023	895	-0.10%
Rome_Tor_Vergata	41.840	12.647	1.33	0.08	0.001	803	0.60%	0.08	0.066	813	-1.00%
**SACOL**	**35.946**	**104.137**	**0.91**	**0.21**	**0.008**	**641**	**1.20%**	**0.23**	**0.197**	**655**	**-3.70%**
SEDE_BOKER	30.855	34.782	0.94	0.11	0.002	773	1.00%	0.11	0.139	784	-1.50%
SERC	38.883	-76.500	1.51	0.06	0.001	820	0.80%	0.06	0.062	826	-0.60%
Saada	31.626	-8.156	0.72	0.16	0.002	729	0.40%	0.15	0.151	749	-1.80%
Sable_Island	43.933	-60.010	1.29	0.04	0.000	881	0.20%	0.04	0.033	876	-0.30%
San_Nicolas	33.257	-119.487	0.94	0.04	0.000	891	0.60%	0.04	0.033	895	-0.10%
Santa_Cruz_Tenerife	28.473	-16.247	0.67	0.14	0.001	755	0.60%	0.12	0.160	790	-1.40%
Sao_Paulo	-23.561	-46.735	1.39	0.09	0.001	778	0.20%	0.09	0.058	784	-0.80%
Saturn_Island	48.783	-123.133	1.34	0.04	0.000	879	1.00%	0.04	0.027	889	-0.20%
Sevastopol	44.616	33.517	1.44	0.07	0.001	811	0.20%	0.07	0.057	814	-0.70%
Sevilleta	34.355	-106.885	1.22	0.03	0.000	896	0.80%	0.03	0.025	904	-0.10%
Shirahama	33.693	135.357	1.24	0.11	0.002	755	0.50%	0.11	0.089	764	-1.10%
Silpakorn_Univ	13.819	100.041	1.32	0.20	0.003	627	-0.30%	0.20	0.115	639	-2.50%
Sioux_Falls	43.736	-96.626	1.33	0.05	0.000	860	0.90%	0.05	0.040	870	-0.30%
Skukuza	-24.992	31.587	1.38	0.07	0.000	814	0.20%	0.07	0.049	820	-0.50%
**Solar_Village**	**24.907**	**46.397**	**0.53**	**0.26**	**0.006**	**625**	**0.20%**	**0.27**	**0.230**	**637**	**-3.10%**
TABLE_MOUNTAIN_CA	34.380	-117.680	1.04	0.02	0.000	918	1.00%	0.02	0.017	926	0.00%
Tabernas_PSA-DLR	37.091	-2.358	1.13	0.07	0.001	826	1.00%	0.07	0.088	836	-0.40%
Table_Mountain	40.125	-105.237	1.38	0.03	0.000	893	0.40%	0.03	0.026	899	-0.10%
**Taihu**	**31.421**	**120.215**	**1.20**	**0.31**	**0.008**	**510**	**-0.80%**	**0.33**	**0.214**	**523**	**-6.00%**
Tamanrasset_INM	22.790	5.530	0.46	0.15	0.003	745	1.00%	0.19	0.248	735	-2.80%
Thessaloniki	40.630	22.960	1.59	0.08	0.001	776	0.10%	0.09	0.060	783	-1.20%
Thompson_Farm	43.109	-70.948	1.47	0.04	0.000	858	1.20%	0.04	0.041	868	-0.30%
Thule	76.516	-68.769	1.49	0.02	0.000	901	1.00%	0.03	0.018	909	-0.10%
Tinga_Tingana	-28.976	139.991	0.83	0.04	0.001	872	3.70%	0.04	0.041	904	0.00%
Tomsk	56.477	85.047	1.44	0.06	0.001	822	0.80%	0.07	0.051	829	-0.60%
Toravere	58.255	26.460	1.41	0.05	0.000	848	0.70%	0.05	0.050	855	-0.30%
Toronto	43.970	-79.470	1.45	0.05	0.000	852	0.70%	0.05	0.041	855	-0.40%
Toulon	43.136	6.009	1.47	0.06	0.000	836	0.70%	0.06	0.050	844	-0.60%
Trelew	-43.250	-65.309	0.80	0.02	0.000	911	1.80%	0.02	0.024	927	0.00%
Trinidad_Head	41.054	-124.151	0.92	0.04	0.000	882	0.60%	0.05	0.034	886	-0.20%
Tucson	32.233	-110.953	1.04	0.04	0.000	894	0.60%	0.04	0.025	899	-0.10%
Tuxtla_Gutierrez	16.755	-93.152	1.45	0.09	0.001	765	0.60%	0.10	0.097	759	-0.80%
UCSB	34.415	-119.845	0.90	0.05	0.000	873	0.70%	0.05	0.037	877	-0.20%
Univ_of_Houston	29.718	-95.342	1.20	0.06	0.000	836	0.50%	0.07	0.051	833	-0.40%

Supplementary Table S1: Continued

Station	Latit.	Longit.	E[α_i]	E[τ_i]	SD[$\tau_{i,d}$]	E[DNI_d]	Δ[DNI_d]	E[τ_d]	SD[$\tau_{d,m}$]	E[DNI_m]	Δ[DNI_m]
Univ_of_Lethbridge	49.682	-112.869	1.29	0.03	0.000	888	0.90%	0.03	0.029	898	-0.20%
USC_SEAPRISM	33.564	-118.118	0.84	0.06	0.000	866	0.30%	0.06	0.035	863	-0.20%
Venise	45.314	12.508	1.53	0.09	0.001	764	0.60%	0.09	0.081	776	-1.90%
Villefranche	43.684	7.329	1.46	0.07	0.001	814	0.70%	0.07	0.065	820	-1.00%
Walker_Branch	35.958	-84.287	1.69	0.06	0.000	812	0.60%	0.07	0.061	814	-0.70%
Wallops	37.942	-75.475	1.55	0.06	0.001	817	0.70%	0.06	0.058	823	-0.50%
Waskesiu	53.917	-106.083	1.44	0.04	0.001	861	1.50%	0.05	0.062	866	-0.10%
WaveCIS_Site_CSI_6	28.867	-90.483	1.25	0.06	0.000	836	0.30%	0.07	0.048	831	-0.50%
White_Sands_HELSTF	32.635	-106.338	1.18	0.03	0.000	893	0.90%	0.03	0.027	902	-0.10%
**XiangHe**	**39.754**	**116.962**	**1.14**	**0.26**	**0.012**	**569**	**0.80%**	**0.29**	**0.305**	**593**	**-9.70%**
Xinglong	40.396	117.578	1.09	0.13	0.008	723	3.10%	0.14	0.155	746	-2.70%
Yakutsk	61.662	129.367	1.58	0.05	0.001	828	2.30%	0.05	0.078	848	-0.60%
Yellowknife_Aurora	62.451	-114.376	1.56	0.04	0.001	841	2.70%	0.05	0.064	858	-0.50%
**Zinder_Airport**	**13.777**	**8.990**	**0.37**	**0.39**	**0.015**	**533**	**-0.40%**	**0.44**	**0.355**	**534**	**-7.90%**
